# Inadequate Gestational Weight Gain Among Saudi Mothers and Pregnancy Outcomes: Riyadh Mother and Baby Follow-Up Study (RAHMA Explore)

**DOI:** 10.3390/healthcare13243258

**Published:** 2025-12-12

**Authors:** Hayfaa Wahabi, Samia Esmaeil, Amel Fayed

**Affiliations:** 1 Research Chair for Evidence-Based Health Care and Knowledge Translation, King Saud University, Riyadh 11451, Saudi Arabia; hwahabi@ksu.edu.sa (H.W.);; 2 Department of Family and Community Medicine, College of Medicine, King Saud University, Riyadh 11451, Saudi Arabia; 3 Department of Family and Community Medicine, College of Medicine, Princess Nourah bint Abdulrahman University, P.O. Box 84428, Riyadh 11671, Saudi Arabia

**Keywords:** gestational weight gain, inadequate weight gain, obesity, prepregnancy body mass index

## Abstract

**Background:** Gestational weight gain (GWG) is commonly used as an indicator of nutritional adequacy during pregnancy and a marker for pregnancy outcomes. This study aims to report the prevalence and extent of GWG inadequacy among Saudi women and to examine the effects of GWG inadequacy on pregnancy outcomes. **Methods**: This study was conducted as part of the Riyadh Mother and Baby Multicenter Cohort Study; it included 6984 women with singleton pregnancies. Adverse pregnancy outcomes—including hypertension, gestational diabetes (GDM), cesarean section (CS), low birth weight (LBW), Neonatal Intensive Care Unit admission (NICU), and macrosomia—were compared between women with adequate and inadequate GWG, based on the Institute of Medicine (IOM) guidelines. **Results**: Of the participants, 2221 women (31.8%) had adequate GWG for prepregnancy BMI, 2959 (42.4%) had inadequate GWG, and 1804 (25.8%) had excessive GWG. Women with normal prepregnancy BMI and inadequate GWG had significantly increased odds of delivering LBW infants (adjusted odds ratio (AOR) = 1.61, 95% CI: 1.17–2.20). Inadequate GWG also decreased the odds of emergency cesarean delivery among women with obesity (AOR = 0.75, 95% CI: 0.56–0.97) and lowered the likelihood of NICU admission for infants of obese women (AOR = 0.59, 95% CI: 0.36–0.97). Women with prepregnancy obesity experienced the highest rate of adverse outcomes; however, the prevalence of all adverse outcomes decreased as the degree of weight gain inadequacy increased. Conversely, underweight women had the highest percentage of LBW, with this percentage increasing as weight gain inadequacy increased. **Conclusions:** The effects of inadequate GWG vary depending on maternal prepregnancy BMI and the specific outcome assessed. For women with obesity, reduced weight gain during pregnancy may be beneficial. In contrast, inadequate GWG is associated with a higher incidence of LBW in women with normal prepregnancy BMI and underweight women.

## 1. Introduction

A healthy pregnancy is characterized by many maternal physiological adaptations that support the developing fetoplacental unit. These adaptations encompass increased maternal blood volume, respiratory and heart rates, and an ongoing increase in maternal weight throughout gestation [1,2].

During the first trimester, maternal weight gain is slight and primarily stems from fat accumulation and early placental development [2]. Conversely, weight gain in the second and third trimesters accelerates and is driven more by fetal growth, the building of maternal fat reserves, and the accumulation of total body water [2]. Overall, the fetoplacental unit—comprising the fetus, placenta, amniotic fluid, and gravid uterus—accounts for nearly 50% of total gestational weight gain (GWG). The remaining gain is split between increased blood volume, extravascular fluid, and breast tissue (25%), and expanded maternal fat stores (25%) [1,2].

The Institute of Medicine (IOM) recommended ranges of GWG based on maternal prepregnancy body mass index (BMI) [2]. GWG is commonly used as a marker of nutritional adequacy during pregnancy and an indicator of pregnancy outcome. Pregnancy complications have been linked equally to inadequate and excessive GWG. Weight gain above the IOM recommendations is associated with increased risk of maternal postpartum obesity and consequent comorbidities such as hypertension (HTN) and type 2 diabetes mellitus [3,4]. In addition to the increased risk of obstetric complications such as pre-eclampsia, gestational diabetes (GDM), cesarean section delivery (CS), the birth of a large-for-gestational-age infant, and macrosomia [5,6,7]. Excessive maternal adiposity has been linked to many offspring diseases, such as childhood obesity, type I diabetes, and adult cardiovascular disease [8,9,10,11]. Although numerous observational studies have documented these significant associations, it remains uncertain whether the relationships between maternal prepregnancy obesity or excessive GWG and childhood outcomes are attributable to direct intrauterine causal mechanisms [12,13]. The relationships observed might also be attributed to environmental factors, lifestyle habits, or genetic traits [14]. Even with thorough adjustments for possible confounding variables in observational research, there may still be persistent confounding issues.

On the other hand, inadequate GWG has been linked to the delivery of low-birth-weight infants, small-for-gestational-age and preterm birth [15,16].

Most studies examining excessive maternal weight and its effects on pregnancy outcomes were published from the Riyadh Mother and Baby multicenter study (RAHMA) database. RAHMA is the largest longitudinal cohort study conducted in Saudi Arabia [17]. Studies from the RAHMA database showed that 60–68% of pregnant women in Saudi Arabia are either overweight or obese before pregnancy [17,18], and that maternal obesity before pregnancy is the primary factor contributing to adverse pregnancy outcomes among this population, including hypertensive disease during pregnancy, GDM, and emergency CS [19,20].

Unlike prepregnancy obesity, excessive GWG was observed in only 24.2% of cases, while inadequate GWG was reported in nearly 50% of Saudi mothers [18].

Most studies investigating maternal weight and its effects on pregnancy outcomes were published from the Riyadh Mother and Baby multicenter study (RAHMA) database. RAHMA is a large cohort study conducted in Saudi Arabia [17]. The study database included pregnancy-related data from 14,568 women and their offspring [17].

To better understand the determinants of maternal weight and its effects on birth outcomes among Saudi women, a series of follow-up studies, named RAHMA (explore), were conducted, including the current study.

The objectives of this study are the following:

1. Report the prevalence and the degree of inadequacy of gestational weight gain among Saudi women.
2. Investigate the effects of the degree of inadequacy of gestational weight gain on the pregnancy outcomes.

## 2. Methods

### 2.1. Study Design and Ethical Approval

This study is derived from database established from the RAHMA (Riyadh Mother and Baby Multicenter Cohort) study, a prospective, multicenter cohort study conducted in three hospitals in Riyadh, Saudi Arabia: King Khalid University Hospital (affiliated with King Saud University), King Fahad Medical City (under the Ministry of Health), and King Abdulaziz Medical City (under the National Guard Health Affairs). Hospitals were selected using a stratified cluster random sampling approach based on hospital type—governmental, military, and university-affiliated.

The original RAHMA study was ethically approved by the Institutional Review Boards (IRBs) of each participating hospital with the following IRB log numbers:

- (Approval No. 11/062);
- (Approval No. 013–017);
- (Approval No. 13–985).

Further to institutional approvals, all participating mothers provided written informed consent after receiving detailed study information in Arabic. Only Saudi nationals were eligible for inclusion. Women with multiple gestations, preterm delivery (<37 weeks of gestation), or missing data on prepregnancy weight were excluded from this analysis.

### 2.2. Participants and Data Collection

All women who gave birth in one of the selected hospitals during the study period, and their newborns, were eligible for inclusion. Data collection utilized a standardized data abstraction form, combining information from maternal and infant medical records with a self-administered maternal questionnaire completed within two days postpartum. This instrument captured demographic, socioeconomic, and lifestyle information, and data from medical records included information about the current pregnancy and antenatal care visits provided to participating women. It included neonatal outcomes such as birth weight, admission to Neonatal Intensive Care Units (NICU), and living status of the newborn.

Exposure and Outcome Measures

1. Prepregnancy body mass index (BMI) was calculated using self-reported weight before pregnancy and height measured at the earliest antenatal visit. BMI categories followed WHO definitions as follows [21]:

- Underweight: If BMI measures ≤ 18.4 kg/m^2^;
- Normal weight: For BMI ranging from 18.5 to 24.9 kg/m^2^;
- Overweight: BMI ranges from 25.0 to 29.9 kg/m^2^;
- Obese: If the BMI is equal to or exceeds 30 kg/m^2^.

Self-reported prepregnancy weight was used to calculate prepregnancy BMI. Self-reported BMI is still the most widely used approach for estimating the prepregnancy BMI, despite the studies indicating underreporting, especially among overweight and obese women [22]. Additionally, much research supports its reliability and validity when reported during pregnancy [23,24,25].

2. Gestational age was assessed using ultrasound-based measures or date of last menstrual period.
3. Gestational weight gain (GWG) was calculated by subtracting the self-reported prepregnancy weight from the mother’s weight at delivery. GWG adequacy was classified based on the IOM predefined ranges. Participants were categorized into three GWG groups: inadequate (less than the IOM range), adequate (within the IOM range), or excessive GWG (exceeded the defined IOM range). IOM defined the ranges of GWG according to the prepregnancy weight BMI as follows [2]:

For women who are prepregnancy underweight (BMI < 18.5), normal weight (18.5–24.9), overweight (25–29.9), and obese (≥30) are recommended to gain 12.5–18 kg, 11.5–16 kg, 7–11.5 kg, and 5–9 kg, respectively, during pregnancy.

Further consideration of the GWG adequacy ratio [26] was derived as a ratio of the observed weight gain over the recommended weight gain in the same gestational duration and expressed as a percentage according to the following Equation (1):

(% Adequacy GWG = (Observed GWG ÷ Recommended GWG) × 100)(1)

Another formula was employed to calculate the recommended GWG, considering the prepregnancy BMI, the gestational age, BMI-specific mean rate of GWG in the third trimester. Equation (2) [19]:

Recommended GWG at the last observed weight measure = ((BMI-specific expected first trimester weight ÷ 13.86 weeks) × (13.86 weeks − GA at first observed)) + ((GA at last weight measure − 13.86 weeks) × BMI-specific recommended mean rate of GWG in the third trimester)(2)

The GWG adequacy ratio was computed as a continuous value and then categorized into four groups: severely inadequate (<70%), moderately inadequate (70% to <90%), adequate (90% to <125%), and excessive (≥125%). The thresholds of <90% and >125% were based on the IOM’s recommended weekly GWG ranges [26].

Primary adverse pregnancy outcomes included the following:

- Gestational diabetes mellitus (GDM) defined according to WHO diagnostic criteria [27].
- Hypertensive disorders of pregnancy (HTN), different disorders of hypertension during pregnancy were defined according to the report of the American National Working Group on High Blood Pressure in Pregnancy. As the incidence of pre-eclampsia, eclampsia and pregnancy-induced hypertension was low, all groups were analyzed as one group [28].
- Macrosomia, defined as birth weight ≥ 4.0 kg [29].
- Low birth weight (LBW), defined as birth weight < 2.5 kg [30].

### 2.3. Statistical Analysis

Categorical variables were summarized using frequencies and percentages. Chi-square was used to test the associations between GWG and categorical variables and Fisher’s Exact test was used if the Chi-square use is not valid. To assess the independent effects of inadequate GWG on selected pregnancy outcomes among different prepregnancy BMI groups, multivariable logistic regression models were fitted. Selection of the pregnancy outcomes and the prepregnancy BMI category was based on the results of the bivariate analysis. Separate regression models were developed: one for LBW among women with a normal prepregnancy BMI, and two others for emergency cesarean section and NICU admission among women with obesity. Covariates such as maternal age and parity were included based on their known relevance. Adjusted odds ratios (AOR) and their 95% confidence intervals (CI) were calculated and reported. A *p*-value < 0.05 was deemed statistically significant. The IBM SPSS Statistics v29 was the statistical software used for all analyses.

## 3. Results

**Figure 1:**
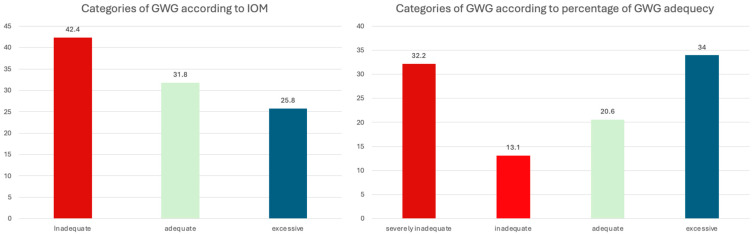
Prevalence of inadequate gestational weight gain (GWG) according to IOM classification and achieved percentage of adequate GWG. GWG: gestational weight gain, IOM: Institute of Medicine.

The study included a cohort of 6984 women with singleton pregnancies. Based on the IOM classification of expected GWG, 2221 women (31.8%) achieved weight gain within the anticipated range for their prepregnancy BMI, 2959 (42.4%) gained less than the expected weight, while 1804 (25.8%) had excessive GWG. Further classification of the participants according to the percentage of expected GWG achieved shows that 1441 (20.6%) achieved adequate GWG, 917 (13.1%) achieved inadequate GWG, while 2252 (32.2%) achieved severely inadequate GWG (Figure 1).

After exclusion of women with excessive GWG, the demographic characteristics of women with adequate and inadequate/severely inadequate GWG are presented in Table 1. No significant differences were observed between the two groups, except for the difference in prepregnancy BMI, where 50% of women classified as obese or overweight experienced inadequate weight gain, while 68.71% and 72.87% of women with normal prepregnancy BMI and underweight, respectively, had inadequate GWG (*p* < 0.01) (Table 1).

Table 2 displays the outcomes of pregnancy for the women who achieved adequate and inadequate GWG based on the prepregnancy BMI. Among women with underweight and overweight BMI, adverse pregnancy outcomes did not differ significantly between those who gain adequate or inadequate GWG. However, women with normal prepregnancy BMI and inadequate GWG had significantly increased odds of having LBW infants; AOR = 1.61, 95% CI: 1.17–2.20). On the other hand, inadequate GWG decreased the odds of emergency CS among women with obesity; (AOR = 0.75, 95% CI: 0.56–0.97), and it lowered the odds of NICU admission for newborns of obese women; (AOR = 0. 0.59, 95% CI: 0.36–0.97) (Table 3).

**Figure 2:**
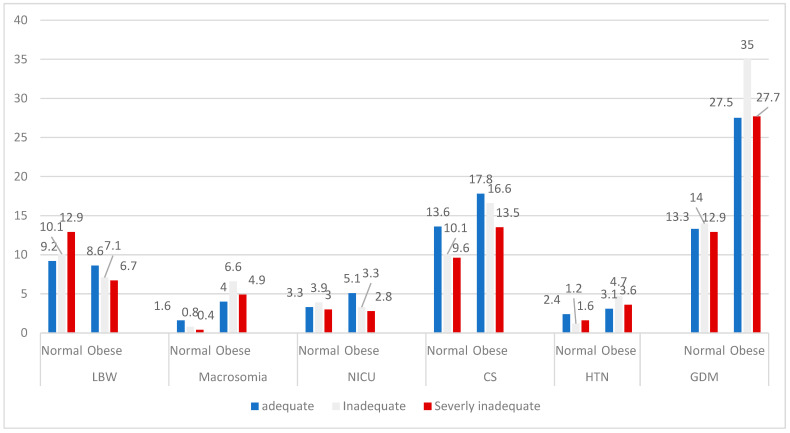
Prevalence of pregnancy adverse outcomes among normal and obese women according to their percentage of adequacy of gestational weight gain. LBW: low birth weight, NICU: neonatal admission to Intensive Care Unit, CS: emergency cesarean section, HTN: pregnancy hypertension, GDM: gestational diabetes.

**Figure 3:**
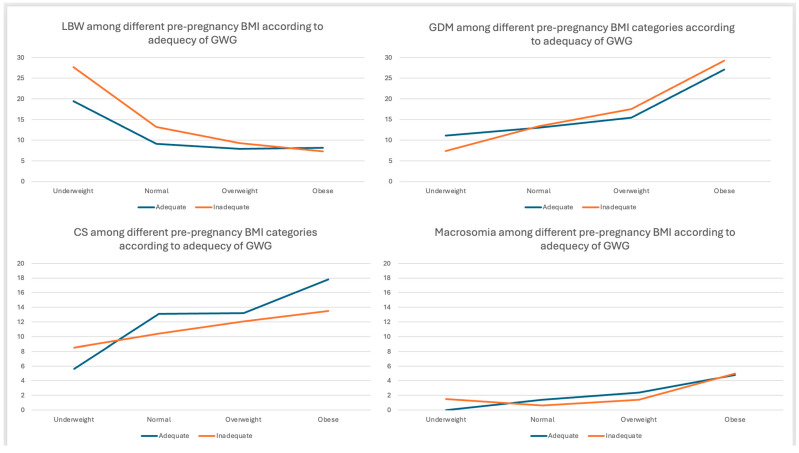
Prevalence of pregnancy adverse outcomes according to prepregnancy body mass index (BMI) and adequacy of gestational weight gain (GWG). GWG: gestational weight gain, BMI: body mass index, LBW: low birth weight, CS: emergency cesarean section, GDM: gestational diabetes.

Further analysis of the impact of GWG inadequacy on pregnancy outcomes indicates a progressive increase in LBW as GWG inadequacy rises among women with normal prepregnancy BMI (Table 4 and Figure 2). In contrast, this relationship is inverse and less pronounced among women classified as obese (Table 4 and Figure 2). Furthermore, the percentage of CS deliveries and NICU admissions in both groups decreases as the percentage of GWG inadequacy increases; however, the prevalence of these outcomes remains higher in the obese group compared to the normal-weight group (Table 4 and Figure 2). The remaining outcomes demonstrated a variable relationship between the severity of GWG inadequacy and the prevalence of outcomes among normal-weight and obese women. Nevertheless, women with prepregnancy obesity experienced the highest percentage of adverse outcomes compared to the other categories of prepregnancy weight, while underweight women had the highest percentage of LBW, with decreasing percentage as prepregnancy weight increases (Table 4 and Figure 3). However, all these differences were not statistically significant.

## 4. Discussion

The findings of this study indicate that over 42% of the mothers in this cohort experienced inadequate GWG according to the IOM guidelines, and that the majority of women with normal prepregnancy BMI and those classified as underweight gained inadequate GWG. The prevalence of inadequate GWG observed in this study aligns with the findings of recent studies conducted in the Gulf region [18,31]. However, it is lower than the prevalence reported in low- and middle-income countries [32] yet higher than the 23% prevalence reported from a meta-analysis of studies encompassing women from diverse ethnic and social backgrounds [5]. The significant variation in the prevalence of inadequate GWG is anticipated due to the impact of numerous modifiable socioeconomic and ethnic factors [32,33].

The demographic profile of the mothers with inadequate GWG did not differ significantly from those with adequate GWG, except for prepregnancy BMI, as shown in Table 1. Other authors reported significant associations between higher levels of education and the younger age group with inadequate or normal GWG [34,35,36]. Modifiable factors, including diet quality and lifestyle, have been linked to a healthy range of GWG [34,37]. A recent study conducted in Saudi Arabia did not find an association between GWG and diet quality; however, the study revealed low consumption of vegetables and fruits across all GWG categories [18]. Our observation of a higher prevalence of inadequate GWG among women with normal prepregnancy weight and those classified as underweight compared to obese women aligns with the findings of previous studies [34,38].

In this study, inadequate GWG is significantly associated with LBW in women with normal prepregnancy weight, as illustrated in Table 2 and Table 3. Nevertheless, the highest percentage of LBW in this study was found among women with prepregnancy underweight (Table 2), with escalating frequency as inadequacy of GWG increases in both groups (Table 3). This finding is consistent with a meta-analysis encompassing over one million women, which also identified association between inadequate GWG and an increased risk of preterm birth, alongside a reduced risk of macrosomia and being large for gestational age [5].

Conversely, the lowest prevalence of LBW was found among obese women, with decreasing frequency as inadequate GWG increases (Table 2 and Table 3). Furthermore, our results demonstrated a consistent association between improved pregnancy outcomes and increased inadequacy of GWG in obese women, with decreased prevalence of CS delivery, NICU admission, and LBW (Table 4). These results align with recent studies, which indicate that maternal and fetal outcomes are improved when weight gain is below the levels recommended by the IOM, or even when weight loss occurs [39], for women classified as obese [38,40,41]. Thus, our findings indicate that the impact of GWG is superimposed upon the more significant determinant of pregnancy outcomes, namely, the prepregnancy BMI.

The association between prepregnancy obesity and adverse pregnancy outcomes has been well-documented by our group and others [20,38,42,43]. A recent observational study conducted in Saudi Arabia demonstrated that maternal prepregnancy obesity was associated with increased prevalence of GDM, hypertensive events in pregnancy, and emergency CS when compared to normal prepregnancy weight [20].

The causal relationship between maternal adiposity and adverse pregnancy outcomes is an interplay of genetic, biological, behavioral, and socioeconomic factors.

Recently published studies have demonstrated the adverse effects of inflammatory biomarkers associated with maternal obesity, such as C-reactive protein and leptin, on preterm birth, pre-eclampsia, and GDM [44]. While the abundant circulating glucose, fatty acids, and lipids in obese mothers cause macrosomia, they may affect the fetal endocrine system and potentially increase the risk of obesity and cardio-metabolic disease in the offspring [45]. Nonetheless, recent studies suggest that the causal relationship between maternal obesity and fetal endocrine and metabolic changes is due to genetic [46], familial, socioeconomic, and behavioral factors [14].

Our findings in this study provide additional evidence regarding the negative impact of being underweight or obese on pregnancy outcomes. They underscore the importance of GWG in influencing these outcomes, as an effect modifier of prepregnancy weight.

### Implications of the Study Results to Practice and Research

1. To address prepregnancy obesity, encourage adequate GWG, and eliminate postpartum weight retention among mothers, it is essential to implement effective interventions that encompass various dietary and exercise modalities as part of maternity care in Saudi Arabia [47,48].
2. Establishing national clinical guidelines for the management of maternal weight during the preconception, antenatal, and postnatal periods is of paramount importance, especially since current IOM guidelines do not adequately address GWG for women who are classified as obese.
3. Given the significant prevalence of obesity and inadequate GWG, along with their adverse outcomes on both mothers and their offspring, it is essential to ensure the provision of adequate infrastructure and personnel to facilitate the consistent implementation of these guidelines.
4. It is imperative to provide health education to Saudi women regarding the potential adverse effects of obesity and underweight on their reproductive health. This educational initiative should be integrated into school and university curricula, given that over 90% of women attend these educational institutions.
5. Future research should focus on examining community-specific interventions aimed at reducing obesity and promoting healthy weight among women of reproductive age.
6. Additionally, investigations should explore the impact of environmental and genetic factors, alongside maternal obesity, on the future health of neonates, children, and adults [49].
7. Future research on maternity obesity and GWG in the Saudi community should adopt a national approach including wide geographical areas to insure generalizability of research outcomes.

## 5. Strengths and Limitations

The primary strengths of our study are the relatively large number of well-documented pregnancies and the high-quality data collected for RAHMA database. In addition, we used a prospective cohort design, which enables us to investigate the causal relationship between multiple outcomes of pregnancy and inadequate GWG. The study provided robust evidence for knowledge gaps in the prevalence and the impact of inadequate GWG among Saudi women. These results can be employed to modify the prepregnancy and antenatal care in the country to fit the needs of the mothers and their offspring for better pregnancy outcomes.

We acknowledge the possible inaccuracy of self-reported prepregnancy weight, which may have affected the calculation of prepregnancy BMI and hence the calculation of GWG. While the study has a large sample size, the data were collected from only one city, which may limit the generalizability to other regions of the Kingdom. Nevertheless, the study benefits from the large pool of participants and the fact that Riyadh represents the largest Saudi population.

## 6. Conclusions

The effects of inadequate GWG differ based on maternal prepregnancy BMI and the specific outcome variable being examined. For women with obesity, reduced weight gain during pregnancy may be beneficial. In contrast, inadequate GWG is linked to a higher incidence of LBW in women with a normal prepregnancy BMI and those who are underweight.

## Figures and Tables

**Table 1:** Demographics of women with adequate and inadequate gestational weight gain.

		Adequate Gestation Weight Gain (N = 2221, 42.9%)		Inadequate Gestation Weight Gain (N = 2959, 57.1%)		*p*-Value
		N	%	N	%	
Age (years)	20–29	1115	43.90	1425	56.10	0.26
	<20	49	38.89	77	61.11	
	30–34	585	42.67	786	57.33	
	35–44	456	40.90	659	59.10	
	45+	16	57.14	12	42.86	
Education	Illiterate	41	37.96	67	62.04	0.20
	School	886	44.68	1097	55.32	
	University and above	640	45.88	755	54.12	
Parity	Primiparous	515	45.22	624	54.78	0.18
	Multiparous	1054	41.98	1457	58.02	
	Grand Multiparous	651	42.58	878	57.42	
Prepregnancy Body Mass Index	Normal	567	31.29	1245	68.71	<0.01
	Underweight	35	27.13	94	72.87	
	Overweight	805	49.94	807	50.06	
	Obese	814	50.03	813	49.97	

**Table 2:** Pregnancy outcomes of adequate and inadequate GWG according to prepregnancy BMI.

		Underweight (<18.5 kg/m^2^)				Normal (18.5–24.9 kg/m^2^)				Overweight (25–29.9 kg/m^2^)				Obese (30+ kg/m^2^)			
		Adequate		Inadequate		Adequate		Inadequate		Adequate		Inadequate		Adequate		Inadequate	
		N	%	N	%	N		N		N		N		N		N	
GDM	normal	32	88.9	87	92.6	489	87.0	1066	86.6	670	84.6	647	82.5	567	72.9	548	70.8
	GDM	4	11.1	7	7.4	73	13.0	165	13.4	122	15.4	137	17.5	211	27.1	226	29.2
	*p*-value	0.50				0.80				0.27				0.36			
HTN	no	35	100.0	93	98.9	559	97.9	1221	98.1	793	98.1	789	98.0	781	95.9	784	96.3
	yes	0	0.0	1	1.1	12	2.1	24	1.9	15	1.9	16	2.0	33	4.1	30	3.7
	*p*-value	0.50 #				0.80				0.82				0.51			
Emerg CS	No	34	94.4	86	91.5	497	86.9	1123	89.6	703	86.8	710	87.9	672	82.2	707	86.5
	Yes	2	5.6	8	8.5	75	13.1	130	10.4	107	13.2	98	12.1	146	17.8	110	13.5
	*p*-value	0.57				0.08				0.51				**0.01**			
LBW < 2500 g	no	29	80.6	68	72.3	520	90.9	1088	86.8	746	92.1	733	90.7	751	91.8	757	92.7
	yes	7	19.4	26	27.7	52	9.1	165	13.2	64	7.9	75	9.3	67	8.2	60	7.3
	*p*-value	0.32				**0.01**				0.33				0.52			
Macrosomia	No	28	100.0	67	98.5	509	98.6	1075	99.4	723	97.6	722	98.6	711	95.2	715	95.0
	Yes	0	0.0	1	1.5	7	1.4	7	0.6	18	2.4	10	1.4	36	4.8	38	5.0
	*p*-value																
Stillbirth	liveborn	36	100.0	93	98.9	568	99.5	1245	99.4	801	98.9	804	99.5	810	99.0	814	99.6
	stillbirth	0	0.0	1	1.1	3	0.5	8	0.6	9	1.1	4	0.5	8	1.0	3	0.4
	*p*-value	0.41 #				0.71				0.27				0.17			
NICU	No	33	91.7	92	97.9	555	97.4	1194	96.1	784	97.5	774	96.1	767	94.6	787	96.7
	Yes	3	8.3	2	2.1	15	2.6	49	3.9	20	2.5	31	3.9	44	5.4	27	3.3
	*p*-value	0.10				0.16				0.11				**0.04**			

GDM: gestational diabetes, HTN: hypertension events during pregnancy, CS: cesarean section, LBW: low birth weight, NICU: Neonatal Intensive Care Unit admission, # Fisher’s Exact test. Significant *p*-value highlighted in bold.

**Table 3:** Odds of LBW (in women with normal BMI) and emergency CS and NICU (in women with obesity) because of inadequate GWG.

	Crude Odds Ratio (95% C.I)	Adjusted Odds Ratio (95% C.I)
Low Birth Weight	1.54 (1.13–2.01)	1.61 (1.17–2.20) *
Emergency Cesarean Section	0.73 (0.56–0.95)	0.75 (0.56–0.97) *
NICU	0.60 (0.37–0.98)	0.59 (0.36–0.97) *

Regression models adjusted for maternal age and parity LBW: low birth weight, CS: cesarean section, NICU: neonatal admission to intensive care unit, BMI: body mass index * *p*-value < 0.05.

**Table 4:** Pregnancy outcomes of adequate, inadequate, and severely inadequate GWG according to prepregnancy BMI.

		Normal (18.5–24.9 kg/m^2^)						Underweight (<18.5 kg/m^2^)						Overweight (25–29.9 kg/m^2^)						Obese (30+ kg/m^2^)					
		90–125% (Adequate)		70–<90% (Inadequate)		<70% (Severe Inadequate)		90–125% (Adequate)		70–<90% (Inadequate)		<70% (Severe Inadequate)		90–125% (Adequate)		70–<90% (Inadequate)		<70% (Severe Inadequate)		90–125% (Adequate)		70–<90% (Inadequate)		<70% (Severe Inadequate)	
		N	%	N	%	N	%	N	%	N	%	N	%	N	%	N	%	N	%	N	%	N	%	N	%
GDM	normal	463	86.7	349	86.0	691	87.1	24	88.9	37	97.4	64	90.1	428	85.3	203	82.5	511	83.2	251	72.5	128	65.0	511	72.2
	GDM	71	13.3	57	14.0	102	12.9	3	11.1	1	2.6	7	9.9	74	14.7	43	17.5	103	16.8	95	27.5	69	35.0	197	27.8
	*p*-value	0.85						0.34						0.54						0.11					
HTN	No	531	97.6	406	98.8	788	98.4	26	100.0	37	97.4	71	100.0	498	98.0	248	97.6	618	98.4	346	96.9	201	95.3	716	96.4
	yes	13	2.4	5	1.2	13	1.6	0	0.0	1	2.6	0	0.0	10	2.0	6	2.4	10	1.6	11	3.1	10	4.7	27	3.6
	*p*-value	0.36						0.27						0.73						0.60					
Emergency CS	No	472	86.4	372	89.9	728	90.4	*25*	*92.6*	*35*	*92.1*	*65*	*91.5*	449	88.2	214	84.3	566	89.7	*295*	*82.2*	*176*	*83.4*	*644*	*86.4*
	Yes	74	13.6	42	10.1	77	9.6	*2*	*7.4*	*3*	*7.9*	*6*	*8.5*	60	11.8	40	15.7	65	10.3	*64*	*17.8*	*35*	*16.6*	*101*	*13.6*
	*p*-value	0.06						0.98						0.08						0.15					
LBW < 2500 g	No	496	90.8	372	89.9	701	87.1	*21*	*77.8*	*30*	*78.9*	*51*	*71.8*	469	92.1	237	93.3	577	91.4	*328*	*91.4*	*196*	*92.9*	*695*	*93.3*
	Yes	50	9.2	42	10.1	104	12.9	*6*	*22.2*	*8*	*21.1*	*20*	*28.2*	40	7.9	17	6.7	54	8.6	*31*	*8.6*	*15*	*7.1*	*50*	*6.7*
	*p*-value	0.07						0.66						0.65						0.51					
Macrosomia	No	488	98.4	369	99.2	698	99.6	*20*	*95.2*	*30*	*100.0*	*51*	*100.0*	459	97.9	234	98.7	568	98.8	315	96.0	183	93.4	663	95.1
	Yes	8	1.6	3	0.8	3	0.4	*1*	*4.8*	*0*	*0.00*	*0*	*0.00*	10	2.1	3	1.3	7	1.2	13	4.0	13	6.6	34	4.9
	*p*-value	0.09						0.14						0.46						0.39					
Stillbirth	liveborn	544	99.6	411	99.3	803	99.8	26	96.3	38	100.0	71	100.0	504	99.0	253	99.6	628	99.5	356	99.2	210	99.5	742	99.6
	Stillbirth	2	0.4	3	0.7	2	0.2	1	3.7	0	0.0	0	0.0	5	1.0	1	0.4	3	0.5	3	0.8	1	0.5	3	0.4
	*p*-value	0.45						0.13						0.49						0.65					
NICU	No	525	96.7	394	96.1	777	97.0	*25*	*92.6*	*37*	*97.4*	*69*	*97.2*	495	97.8	248	97.6	605	96.3	*338*	*94.9*	*202*	*96.7*	*721*	*97.2*
	Yes	18	3.3	16	3.9	24	3.0	*2*	*7.4*	*1*	*2.6*	*2*	*2.8*	11	2.2	6	2.4	23	3.7	*18*	*5.1*	*7*	*3.3*	*21*	*2.8*
	*p*-value	0.71						0.51						0.28						0.17					

GDM: gestational diabetes, HTN: hypertension events during pregnancy, CS: cesarean section, LBW: low birth weight, NICU: Neonatal Intensive Care Unit admission. Numbers in italic indicate increasing or decreasing trend in prevalence.

## Data Availability

Data from this study are available to researchers upon request and approval of the Institutional Review Board at King Saud University. The request and approval of data sharing are independent of the research team.
